# Multispeciality Approach in the Management of Patient with Hereditary Gingival Fibromatosis: 1-Year Followup: A Case Report

**DOI:** 10.1155/2010/575979

**Published:** 2010-12-23

**Authors:** T. Ramakrishnan, Manmeet Kaur

**Affiliations:** Department of Periodontology and Implantology, Meenakshi Ammal Dental College and Hospital, Maduravoyal, Chennai 95, India

## Abstract

*Background*. Hereditary gingival fibromatosis is a fibrotic enlargement of the gingiva. It may exist as an isolated abnormality or as part of multisystem syndrome. This paper reports a case of 16-year-old male with generalized severe gingival overgrowth, involving the maxillary and mandibular arches and covering almost all teeth. *Methods*. Periodontal management of gingival enlargement included gingivectomy in both arches except in the lower right molar region where flap surgery was done under general anesthesia. After a 2-month followup period, orthodontic treatment was started with fixed appliances. Monthly periodontal checkups and maintainance (scaling and polishing) were scheduled to control the gingival inflammation. *Results*. Reevaluation of the patient of surgical treatment after two months did not show any recurrence of condition; however, minimal overgrowth was noted 1 month after the beginning of orthodontic treatment which was treated nonsurgically. *Conclusions*. Although the risk of recurrence is high with this condition, surgical treatment with correction of malocclusion and regular followup can provide excellent outcome as seen in this case.

## 1. Introduction

Hereditary gingival fibromatosis (HGF) is a rare benign, nonhemorrhagic fibrous enlargement of gingival tissue [1]. Males and females are equally affected at a phenotype frequency of 1 : 750,000 with varying intensity and expressivity even in individuals within the same family [1, 2]. The disease may be found as an autosomal dominant or autosomal recessive mode of inheritance [2–4]. 

The hyperplastic gingiva usually presents a normal color and has a firm consistency with abundant stippling. The gingival enlargement usually coincides with the eruption of the permanent dentition although it may occur during the eruption of primary dentition or rarely at birth. It may be localized (nodular) or generalized (symmetric), thus potentially interfering with speech, closure of the lips, and mastication resulting in both aesthetic and functional problems [2, 5]. 

 HGF is usually seen as an isolated disorder, but it may also develop as one feature of several rare multisystem syndromes such as Zimmerman-Laband (ZLS), Jones, Ramon and Rutherford syndrome, Juvenile hyaline fibromatosis, systemic infantile hyalinosis, and mannosidosis. HGF has been recorded in association with hypertrichosis, mental retardation, epilepsy, progressive sensorineural hearing loss, and abnormalities of extremities, particularly of fingers and toes [6]. 

Conditions associated with Zimmerman-Laband syndrome are gingival fibromatosis, abnormal fingers, fingernails, nose, and ears. Other findings associated with ZLS are Splenomegaly, hepatomegaly, and hyperextensible metacarpophalangela joint [7]. In Jones syndrome, gingival fibromatosis associated with progressive sensoneural hearing loss was found [8]. In Ramon syndrome, the findings were gingival fibromatosis, cherubism, seizures, mental deficiency, hypertrichosis, stunted growth, and juvenile rheumatoid arthritis [9]. In Rutherford syndrome conditions associated with gingival hypertrophy include corneal opacity, mental retardation, failure of tooth eruption, and aggressive behavior [10]. In juvenile hyaline fibromatosis, gingival fibromatosis was associated with multiple subcutaneous tumors, dysseborrhea, sclerodermiform atrophy, whitish nodules, and osteolytic and osteoclastic skeletal lesion [11]. In systemic infantile hyalinosis, gingival fibromatosis was associated with thickened skin, focal skin nodularity, restricted movement, joint contractures, and osteoporosis and most of the individuals fail to thrive [12]. In Mannosidosis, gingival hypertrophy was associated with deafness, muscle hypotonia, craniofacial dysmorphism, mental retardation, and Immunoglobulin deficiency [13].

## 2. Case Report

A 16-year-old male presented at the Department of Periodontology and Implantology, Meenakshi Ammal Dental College and Hospital, Chennai, with the chief complaint of gingival swelling covering both mandibular and maxillary teeth. The swelling caused difficulties in speaking and eating, and he also had obvious implications for his aesthetic appearance. The patient's medical history appeared to be noncontributory to the development of the gingival enlargement. The patient in this report had no history of using drugs such as phenytoin, nifedipine, or cyclosporine. The patient revealed a family history which was apparently significant to the present finding. His maternal grandfather and maternal uncle had similar gingival enlargements but were deceased. The patient's mother (58 years old) (Figure 5), maternal aunt (59 years old), and sister (26 years old) (Figure 6) had similar gingival enlargement involving, to various extents, the maxilla as well as the mandible, which was treated at various points of time.

**Figure 1:**
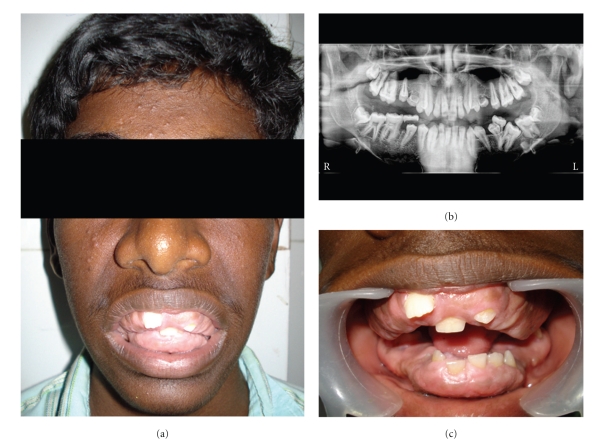
(a) Showing patients photograph. (b) OPG. (c) Intraoral front view.

**Figure 2:**
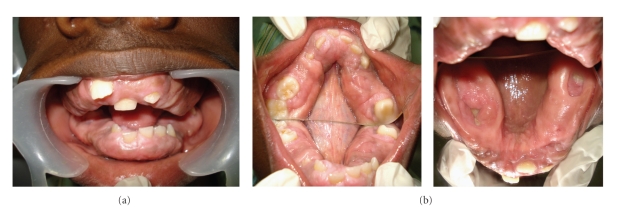
(a) Preoperative intraoral front view. (b) Preoperative mirror image of lower arch and upper arch (Intraoral).

The intraoral examination revealed generalized, severe gingival overgrowth involving both the mandibular and maxillary arches (Figures 1(a), 1(c), 2(a), and 2(b)). The gingival overgrowth was seen as firm, dense, fibrous, and painless enlargement with normal gingival color. Panoramic radiographic examination revealed complete permanent dentition with retained deciduous molars (54, 64, 75, and 85) (Figure 1(b)). Teeth were malaligned with minimal alveolar bone loss. In the light of patient, family history, and these clinical observations, a provisional diagnosis of HGF was given based on the family history and clinical examination.

## 3. Treatment

**Figure 3:**
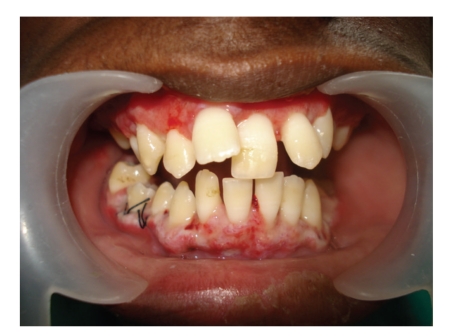
Postoperative intraoral view after 2 weeks.

**Figure 4:**
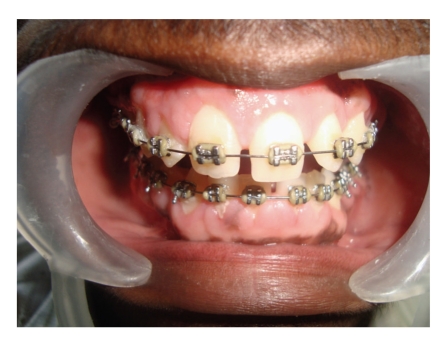
Orthodontic treatment showing excellent improvement after 10 months.

**Figure 5:**
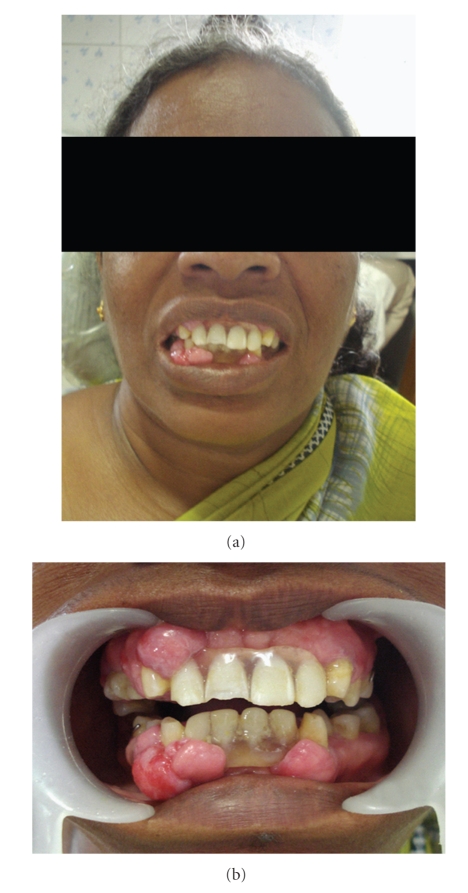
Showing mothers photograph and intraoral front view.

**Figure 6:**
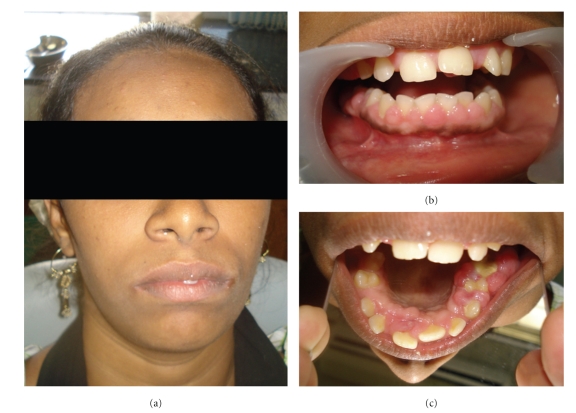
Showing patients sisters photograph, intraoral front view and upper mirror image.

Functional and esthetic disability indicated a need for surgical intervention which was carried out under general anesthesia after informed consent was obtained from the patient's parent. The treatment consisted of an external bevel gingivectomy at all quadrants using scalpel and electrocautery (Colorado tip) with total excised tissue weighing approximately 160 g. Postoperative bleeding did not occur. The deciduous molars were removed at the time of surgery, as they were held in place only by the bulk of gingiva. Postoperatively, the patient was advised to continue the antibiotic (amoxillin-500 mg tds) for 5 days, analgesic (ibuprofen 400 mg) as and when needed and to use 0.2% chlorhexidine digluconate mouth rinse for 2 weeks postoperatively (Figure 3). The patient was recalled for checkup at 1, 3, and 6 weeks intervals postoperatively. The postsurgical healing was excellent as the patient maintained good oral hygiene. Orthodontic treatment was started after two months and she is still in progress (Figure 4).

## 4. Histopathological Examination

The fixed tissue specimens (10% buffered formalin) showed highly fibrous connective tissue with dense collagen bundle arranged in haphazard manner with moderate number of spindle-shaped fibroblasts. Focal areas of chronic inflammatory cell infiltrate with lymphocytes, plasma cells, neutrophils, and few mast cells were seen. Blood capillaries showed compressed lumen and some were engorged with RBCs. The overlying epithelium appeared hyperkeratotic stratified squamous of variable thickness with irregular rete ridges showing hyperplasia in some areas and atrophy in others. Superficial layers of epithelium showed features of edema.

## 5. Discussion

HGF may be found as an autosomal dominant or autosomal recessive mode of inheritance with variable penetrance and expressivity [2–4]. The mode of genetic transmission in this patient points to an autosomal dominant gene because family members of both sexes were affected, and the condition was present in successive generations (grandfather, mother, and children). The enlargement began with the emergence of deciduous dentition and gradually increased to cover the teeth completely, delaying the exfoliation of primary molars. Patient exhibited a more common generalized (symmetric) gingival enlargement. Severe growth resulted in crowding of underlying teeth, speech impediment, difficulty with mastication, and prevented normal closure of lips. Syndromic abnormalities commonly seen in association with HGF were not observed in this patient. To date, three different loci are associated with isolated form of HGF: two map to chromosome 2 (GINGF on 2p21-22 and GINGF3 on 2p22.3-p23.3) and one maps to chromosome 5 (GINGF2 on 5q13-HGF1 locus 2p13-p21) [2, 14]. Of these loci, only the SOS1 (*son-of-sevenless-1*) gene that codes for guanine nucleotide exchange factor for *ras* proteins has been described [15, 16]. 

The histopathologic features observed in the present case had the typical appearance of the gingival lesions in HGF, and the provisional diagnosis was confirmed. Although the mechanism that leads to the accumulation of abnormal amounts of gingival tissue in HGF is still unknown, there is some evidence that certain defects may lie in the anabolism of gingival tissue products. 

HGF cannot be cured but may be controlled with varying degree of success. The best time to initiate treatment to HGF is when all of the permanent dentition has erupted because the risk of recurrence is higher before it. Treatments vary according to the degree of severity of gingival enlargement. When the enlargement is minimal, thorough scaling of teeth and home care may be sufficient. However, excessive gingival tissue and esthetic and functional impairment dictate the need for surgical intervention [2, 5]. Because of the severity of the involvement with no attachment loss and pocketing in this case, an external bevel gingivectomy was the favored treatment. In areas with inadequate attached gingiva (right lower posterior region), flap surgery was carried out. Psychological benefits because of cosmetic improvement outweigh the probability of recurrences in such severe cases. In the present case, because of periodic appointments with good plaque control measures with appropriate and timely orthodontic treatment, recovery is expected to be uneventful. A multispeciality approach involving a periodontist, orthodontist, oral surgeon, and oral pathologist helped to provide a successful treatment in this case. Recurrence of gingival over growth in HGF is not uncommon. Therefore, more frequent followup might be required.
